# Novel CE-CBCE feature extraction method for object classification using a low-density LiDAR point cloud

**DOI:** 10.1371/journal.pone.0256665

**Published:** 2021-08-25

**Authors:** Muhammad Rabani Mohd Romlay, Azhar Mohd Ibrahim, Siti Fauziah Toha, Philippe De Wilde, Ibrahim Venkat

**Affiliations:** 1 Department of Mechatronics Engineering, International Islamic University Malaysia, Jalan Gombak, Kuala Lumpur, Malaysia; 2 University of Kent, Canterbury, United Kingdom; 3 School of Computing and Informatics, Jalan Tunku Link Gadong, Universiti Teknologi Brunei, Bandar Seri Begawan, Brunei Darussalam; Newcastle University, UNITED KINGDOM

## Abstract

Low-end LiDAR sensor provides an alternative for depth measurement and object recognition for lightweight devices. However due to low computing capacity, complicated algorithms are incompatible to be performed on the device, with sparse information further limits the feature available for extraction. Therefore, a classification method which could receive sparse input, while providing ample leverage for the classification process to accurately differentiate objects within limited computing capability is required. To achieve reliable feature extraction from a sparse LiDAR point cloud, this paper proposes a novel Clustered Extraction and Centroid Based Clustered Extraction Method (CE-CBCE) method for feature extraction followed by a convolutional neural network (CNN) object classifier. The integration of the CE-CBCE and CNN methods enable us to utilize lightweight actuated LiDAR input and provides low computing means of classification while maintaining accurate detection. Based on genuine LiDAR data, the final result shows reliable accuracy of 97% through the method proposed.

## Introduction

A LiDAR sensor provides a solution for mobile applications where the system needs to be compact, lightweight and handy [1, 2]. It has a 360° degree field of view [3], possesses high accuracy of distance measurement and in contrast to a camera, it does not depend on the light intensity of the surroundings [4, 5]. The LiDAR sensor is robust to illumination variation [6], and can be used to obtain the transformation matrix between 2D coordinate system and 3D model of the scene [7]. Its detection range is also comparatively higher in accuracy and provides better reliability when compared to stereo methods [8, 9].

In some applications where portability and mobility are of prime importance, a single sensor which acts as the detection system is required [10, 11]. Examples of such applications are mobile monitoring robots [12], robotic navigation aids [13], skateboarder near-crash identification [14], smart canes for blind people and traffic monitoring for electric-assisted bicycles (e-bikes) [15]. Hence, there is a demand for system navigation by using a single portable 3D sensor [16].

## Related works

An integral parts of robotics [17–20], object recognition with LiDAR sensor commonly depends on feature extraction for classification of objects. Accurate object detection and classification allows object tracking, road signs detection [21], scene understanding [22] and behaviour recognition [23]. 3D LiDAR traits based on local surface and key-points are amongst the main features for extraction within object recognition [24]. These key-point detection algorithms detect variances, normal vector, curvature or any other spatial geometric attributes as its feature extraction objectives. Following transformation, these key-points are examined for matches between predicted object and newly identified object. However, this does result in a significant increase in computational cost and time as the feature extraction solely depends on finding neighboring surrounding and determining adjacent points [25].

Other researchers suggested usage of histograms based on key-points to gather spatial features of point clouds in varying dimensions. The authors of [26] measured orientation angles in between points and their neighbors to create a histogram which is based on feature descriptors; namely fast point feature histogram (FPFH). Despite the FPFH showing promising results in terms of processing timing, the neighboring space of the FPFH descriptor is still considerably broad, resulting in a large execution time.

Yamada et al. [27] present a gait-based human identification using a real-time multi-line LiDAR. The author combines LiDAR data with long short-term memory for gait recognition with different appearances. Even though it performs well for on face recognition and human identification, gait-based feature extraction is mostly suitable for biometric recognition. Hence, limiting its object of detection from other objects in the environment.

The authors of [28] proposed a 3D convolutional kernel at varying scales to derive the features of targeted objects with distinct resolutions. Hence, the positional structure of the point clouds can be assembled in a more particular manner in which object classification accuracy, semantic recognition and other applications in the sequence are enhanced. However, the requirements of revising new points in a tree-based storage construction are high, specifically where point clouds were collected incrementally along the process.

In contrast to spatial feature extraction from individual points, clusters of points can be considered as voxels to be processed as coarse feature extraction. In [29], the authors suggested a semantic classification, partitioning spatial space into organized fixed-sized voxels. This solves the issue of processing difficulties within uneven density distributions. However, voxels with fixed size segregation may be inadequate for object recognition because of the unsymmetrical framework of the point clouds and its densities.

The authors of [19] computed a feature vector for pedestrian recognition with 195 collected features including 2D covariance matrix, normalize 2D histogram and slice feature. Tian et al. [30] proposed multiple feature extraction which consists of 9 features including density, centroid and variance. However, the features are from a global voxel are made from the entire LiDAR’s point of view thus contributing to heavy computation. This is mainly due to calculation cost is proportion to the number of voxels rather than the number of points [31]. Choi et al. [32] proposed using the Region of Interest (ROI) method with smaller number of features extracted. Even though only basic features are extracted such as width length and height, it does require an eagles-eye point of view by an airborne LiDAR or integration of numerous numbers of LiDAR’s for accurate scanning.

A few researchers chose bottom-up or top-down approaches to extract information and distinguish objects, however they do not consider the variation of point densities which will cause significant change of accuracy [33]. This is due to density changes with the distance from the LiDAR [34]. Especially in a very sparse LiDAR data recognition performance drastically decrease as distance between human and LiDAR increases, due to the number of points being inversely proportional to the square of the distance between a human to LiDAR [35].

An object detection network which views data in the form of a matrix is proposed by [36], with continuous properties from channel views as its extracted features and 2D convolution network. However, the approach requires LiDAR’s with higher density to feed channels of scanning for extraction. Due to disparity in object detection using sparse LiDAR point cloud, there is a necessity to develop a method which could perform well with such limited input. Existing works mainly provide solutions for high density point clouds, often involving heavy computational cost and computing load. Alternative methods which work on low density data are commonly limited to binary classification and insubstantial when dealing with multiple class detection. Table 1 shows the findings of main comparison feature extraction methods.

**Table 1:** Summary of the findings of selected existing works.

Ref	Method	Extracted features	Dimensional count	Class of Object	Results	Remarks
[30]	Multiple feature extraction (MFE)	Point count (*N*), point density (*ρ*), voxel centroid (*μ*), point variance (*σ*^2^), point covariance (${\bar{\sigma }}^{2}$), point eigenvector (*ν*), point eigenvalue (*γ*), surface curvature (*k*) and divergence degree (*F*)	27	Bush, Tree, Pedestrian, Pole, Wall	Accuracy of 92.84%	Tested with varying machine learning algorithm, with less comparison with other feature extraction method. Comparison with our proposed method is shown in results section.
[19]	Feature vector (FV)	2D covariance matrix in 3 zones, 2D histogram for x-y plane and 2D histogram for y-z plane	175	Pedestrian	True positive rate is increased approximately 0.15 and 0.1 from classifier trained by SVM.	Deals with high dense point cloud data, high computing load for mobile robot usage
[32]	Region of interest (ROI)	Width (*w*), length (*l*), height (*h*), width difference (Δ*w*) and length difference (Δ*l*)	5	Ground Classification	Filter out the amount of unwanted raw data for the actual tracking. Introduce feature-based Object geometry for precise estimation of the system state. Average processing time of 20ms.	Limited feature extracted, would be tough to differentiate classification of numerous subjects due to indistinct extracted value.
[37]	Depth Map (DM) method	RGB images (using monocular camera), depth maps (or range view) and 3D point clouds	3	Pedestrian, Vehicle (cars, vans and trucks), Cyclist	Average F1-score of 96.62%	Involving fusions of two main sensors which is the monocular camera and LiDAR.
[38]	Distance Dependent method feature extraction	Max height, height, density, intensity, binary and Multichannel max height voxels:	6	Cars, pedestrians and cyclists.	These changes lead to improvements, most notably of 2.7% accuracy percentage on the 0-35meter range for easy category and 5.0% on the 35–70 meter range for hard category.	Involving high density dataset taken from Velodyne 64 channel LiDAR sensor.

### Contributions

It is vital to develop a classification method which can work with a sparse 3D point cloud, while providing enough leverage for the classification process to accurately recognize objects within limited computing capacity. Thus, this paper proposes an object recognition system with multiple feature extraction based on segregated clusters from LiDAR point clouds. To extract geometry features, we rasterize each point cloud of the object in a local voxel slice model based on its centroid.

The method proposed introduces local abscissa, ordinate and applicate (z-axis) voxels to reduce the computational cost of computing global voxels in the spatial domain while removing uncertainties of varying densities, discarding rigid transformation and unsymmetrical structure of point clouds associated with global voxel with arranged fixed-sized segregation. This research further introduces a novel feature extraction technique which considers a collection of features viz., density to centroid height ratio and density to volume ratio. These features capture the point cloud disparity and achieves a higher detection rate when compared to other state-of-the-art feature extraction methods. The proposed feature extraction helps to overcome the inconsistent point cloud detection due to the single point of view in scanning, allowing accurate object recognition from using a single actuated LiDAR sensor.

Employing machine learning for object classifiers has been a major interest of researchers as a means to train extracted features including for LiDAR point cloud classification [39, 40]. Bobkov et al. [41] implement a convolutional neural network (CNN) with 5 filters and pooling for layer extraction. Whereas Tian et al. [30] implemented multiple object features with annotated labels incorporated with an initialized neural network. Considering the success of machine learning algorithms in various areas including feature-based object classification [42], this research further optimizes the features extracted from the proposed method to be trained with selected machine learning optimizers. The algorithms selected are the k-nearest neighbor (k-NN), decision tree (DT) and convolutional neural network (CNN).

For the class of object detection, static object classification has been shown with target-level and with low level data [43]. In this paper, we will focus on methods addressing moving road users in conventional streets. We selected three important class of objects detection, categorically pedestrians, motorcyclists and cars. These three objects are typical on-road scene [44, 45]. Thus, its detection provide critical information for security and surveillance, law enforcement monitoring, search and rescue team [46, 47].

To the best knowledge of the authors, no existing work in the field of machine vision explicitly exploits information from a single unit, sparse LiDAR sensor for 3D scanning to achieve object recognition with high accuracy rates. We prove that object recognition can be obtained using a single unit, single stripe, actuated LiDAR with low computing necessity via the fusion of Clustered Extraction and Centroid Based Clustered Extraction (CE-CBCE) methods to accomplish high-reliability object recognition from sparse LiDAR point cloud data. To summarize, our main contributions are

- State-of-the-art combination of the Clustered Extraction (CE) and Centroid Based Clustered Extraction (CBCE) method which includes features extracted from the abscissa, ordinate and applicate voxels, a novel density to centroid height interval ratio and density to volume ratio. These features of sparse LiDAR point cloud data allow accurate classification from a single detection sensor.
- Result analysis and comparison of the CE-CBCE method trained by using k-NN, DT and CNN classification methods. The CE-CBCE optimized with the CNN classification recorded the best accuracy, excellent and consistent scores in terms of recall, precision and F1-score. The results show that the proposed method outperforms other state of the art feature extraction methods.
- Genuine 3D LiDAR point cloud data taken from a custom-built mobile robot with a detection system from a single LiDAR sensor. The data consist of 1200 scans of 3 main objects classes with 4 pose orientation headings. The data have been made public and can be accessed accordingly [48].

The rest of the paper is organized as follows. Section II introduces the technical issues and proposed methods for gathering and processing data from the LiDAR sensor. Here, the description of each step is explained in detail. Section III presents the results and analysis of the output from our experiment. Finally, conclusions and future recommendations are discussed in Section IV.

## Proposed method

Primarily the research intends to propose a novel clustering-based feature extraction technique to exploit discriminative features from the scarce LiDAR point cloud data. Initially, the process starts with background filtering and clustering the raw point cloud data. Then the proposed method extracts features from the clustered object point clouds. The proposed method is divided into two parts namely Clustered Extraction (CE) and the Centroid Based Clustered Extraction (CBCE) method. Through this method, there are less computational power required when compared to using global voxels within the spatial domain. The final stored elements were taken from sparsely distributed values in the LiDAR point cloud, which will then be trained by selected classification methods (k-NN, DT and CNN) for human detection. A written consent of this research which involves detection of human subjects have been approved by IIUM Research Ethics Committee (IREC) with ID No: IREC 2017–066.

The following section explains the details of each procedure step by step. The sequence starts with data collection, filtering, clustering and finally object classification.

### Data collection

**Fig 1:**
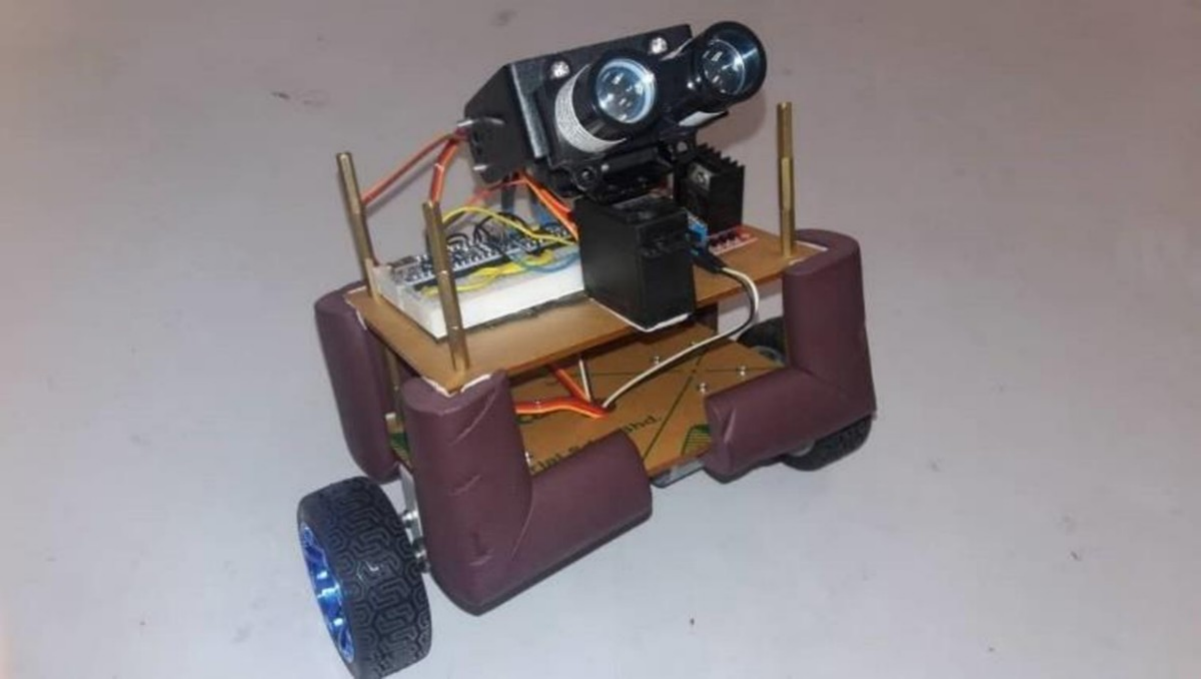
Mobile robot prototype with a single actuating LiDAR sensor for object recognition.

For genuine data collection purposes, we have constructed a mobile robot with LiDAR based sensor. The hardware components include Garmin LiDAR Lite v3, Arduino Uno, FS5109 servo motor, L298N motor driver. For wireless communication, XBee which comes readily with TX and RX communication modules, allowing wireless data transmission [49] and Li-Po external battery are required, providing mobility to the system. The scanning degree is fixed at 130°, resembling a human’s point of view [50]. The mobile robot scanning can be seen in Fig 1.

Over 1200 scans have been collected which contained 400 objects for each of the three cluster categories viz., human, motorcyclist and cars. These three classes of objects are the most commonly found for on-road scene. For comparison purposes, we have selected the same number of samples for each class and orientation. The scenes are recorded within indoor and outdoor environments, during day and night for better reflection of the real-world environment. The position of the object detected varies with distance up to 40 meters from the mobile robot. This is the effective distance of the proposed method and the range of detection for the LiDAR sensor. The clustered point clouds are classified into 3 categories as mentioned before.

**Fig 2:**
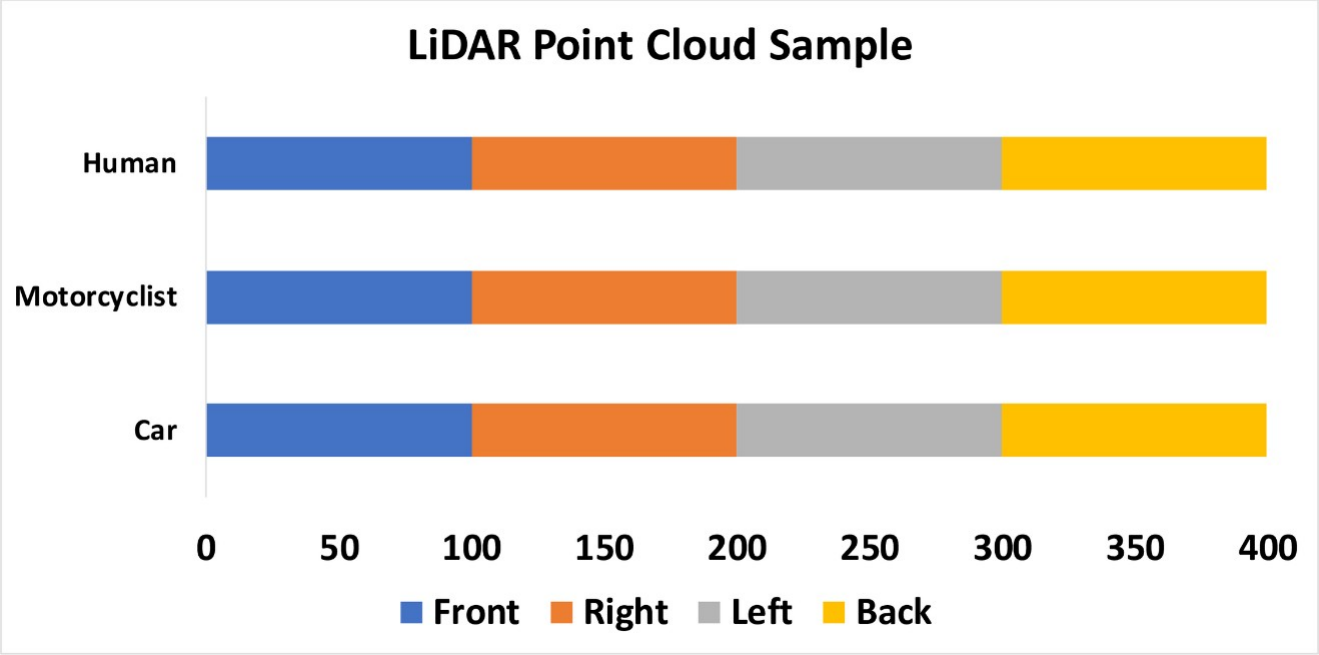
No of point cloud samples for each class and its pose orientation.

However even for the same object, different poses (rotations as well as translations) with respect to the LiDAR could result in different coordinates (ex: varying x/y/z minimum and maximum, object centroid and volume size). Therefore, we have taken the sample with various distance from 1 m to 40 m, with different pose orientation of the object from front, right left and back side of the targeted sample. Fig 2 below shows the total number of samples across the 3 class of object detection and its orientation facing the mobile robot.

### Filtering

From the raw point cloud data collected with the mobile robot, unnecessary noises are removed from the scene. A threshold value of 500 *z* coordinate is fixed, approximately 5 meters from the ground. The points above the threshold are considered as non-disturbance. It does not pose as an obstacle for the mobile robot movement and does not represent any classes from the targeted class of object detection. Therefore, all points which surpass the set threshold value are subtracted from the point cloud, before entering the clustering process.

### Clustering

**Fig 3:**
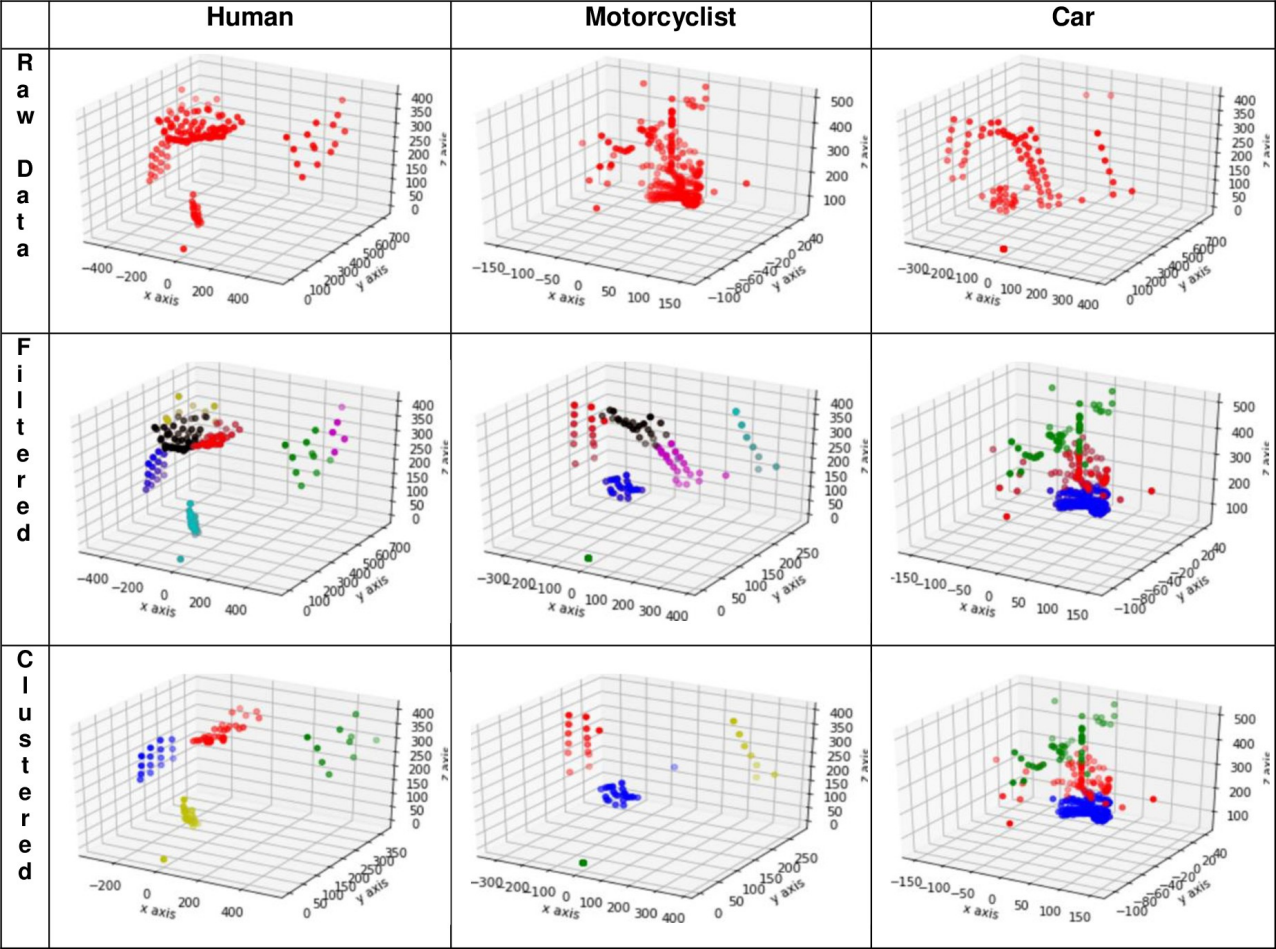
Point cloud data example of human, motorcyclist and car subject showing raw data, point cloud following filter process and point cloud post clustering.

Following filtering, the remaining point cloud goes through the process of clustering with the k-means clustering algorithm. Fig 3 shows raw, filtered and clustered data of all subjects of recognition.

Up until this point all classification methods go through the same scanning, filtering and clustering procedures. In the next step of feature extraction, the proposed technique will be compared with other state-of-the-art classification method. To solely compare the performance of each feature extraction method, we have designed the process to be non-end-to-end classifiers with pre-processing procedures (filtering & clustering) and post-processing steps (classification methods using DT, k-NN and CNN).

### Clustered Extraction (CE) method

The LiDAR point cloud gives output in Cartesian coordinate system with the x, y & z origin set to be the position of the LiDAR on top of the mobile robot.

The first part of our proposed clustered extraction (CE) method is the feature extraction of α, β, γ. Given a point cloud,

p=[x,y,z] (1)

the CE method proposed is explained below as,

P=[α,β,γ] (2)

where P is the clustered LiDAR point cloud data, extracted into three parts. The first part is denoted by alpha *α*, which stores the values of width (*w*), length (*l*), height (*h*) of the object, and the number of points in the cluster (*N*). The second part is represented by the array beta *β* which stores the number of elements within the segregated intervals represented as *x*_*dataset*_, *y*_*dataset*_ and *z*_*dataset*_. The unique feature of *β* is the number of points derived from abscissa, ordinate and applicate voxels. The third part is the minimum and maximum value of each axis in the clusters, denoted by gamma *γ*.


α=[w,l,h,N] (3)

$$\beta =[x_{dataset},y_{dataset},z_{dataset}]$$ (4)

$$\gamma =[x_{min},x_{max},y_{min},y_{max},z_{min},z_{max}]$$ (5)

From Eq (1), we compute the centroid of the cluster *c*,

$$c=\left[\frac{x_{1}+x_{2}+\cdots x_{n}}{N},\frac{y_{1}+y_{2}+\cdots y_{n}}{N},\frac{z_{1}+z_{2}+\cdots z_{n}}{N}\right]$$ (6)

Centroid *c* acts as the origin of the local voxels for each cluster. From the centroid, addition for voxel borders is constructed with a predetermined increment value. These will act as the abscissa, ordinate and applicate voxels. Therefore,

$$\begin{array}{l} x_{\mathrm{dataset}}=[\left|x_{c}|,|x_{c-\Delta }|,\ldots |x_{\mathrm{floor}}\right|,\\ \;\left|x_{c}|,|x_{c+\Delta }],\ldots |x_{\mathrm{ceiling}}\right|]\\ y_{\mathrm{dataset}}=[|y_{c}|,|y_{c-\Delta }|,\ldots |y_{\mathrm{floor}}|,\\ \;\left|y_{c}|,|y_{c+\Delta }|,\ldots |y_{\mathrm{ceiling}}|\right] \end{array}$$

and

$$\begin{array}{l} Z_{\mathrm{dataset}}=[|z_{c}|,|Z_{c-\Delta }|,\ldots |Z_{\mathrm{floor}}|,\\ \;\left|Z_{C}|,|Z_{c+\Delta }|,\ldots |Z_{\mathrm{ceiling}}\right|] \end{array}$$ (7)

where |.| indicates cardinality. The initial value of the dataset interval is denoted as *x*_*floor*_; where *x*_*floor*_ presents the value of *x*_*min*_ rounded down to increment value Δ (in this case we set it to be 50),

$$x_{floor}=\left\lfloor \frac{x_{min}}{\Delta }\right\rfloor \times \Delta$$ (8)

Finally, the end value of the dataset is defined as *x*_*ceiling*_, which is the *x*_*max*_ value rounded up to the nearest hundred,

$$x_{ceiling}=\left\lfloor \frac{x_{max}}{\Delta }\right\rfloor \times \Delta$$ (9)

The stored elements of maxima and minima of the coordinate together with the number of elements within a determined interval serve as the input for the human detection classifier. The same procedures are done to acquire ceiling and floor value of *y*_*dataset*_ and *z*_*dataset*_.

### Centroid Based Clustered Extraction (CBCE) method

From here onwards, two additional collective features are extracted from the point cloud denoted by delta *δ* (for features related to density to centroid height ratio (ρh) and epsilon *ε* (for features related to density to volume ratio (ρV). First, the collective features of *δ* are discussed.


δ=[h,ni−1h,nih,nch,ρi−1c,ρic,ρidiff,[ρh]i] (10)

Initially, the height of the object ***h*** is determined and it is divided by the total number of parts ***t*** set to be 10 as default to acquire height of *i*-th part of n ${(n}_{i}^{h})$. For each part, the centroid height is calculated as a reference point.


$$n_{i}^{h}=\frac{h}{t}+n_{i-1}$$ (11)

For *i* = 1,2,…*t*. Height of cluster *n*, ${(n}_{i}^{h})$ is calculated in (12).


$$n_{c}^{h}=\frac{n_{i}^{h}-n_{i-1}^{h}}{2}+n_{i-1}^{h},$$

$$for\;[a,b)=[a,b[=\{n_{c}^{h}\in R|a\leq n_{c}^{h}<b\}$$ (12)

For $n_{i}^{h},n_{i+1}^{h}\ldots n_{t}^{h}$. Then density between interval is denoted by $\rho_{i}^{diff}$ acquired through subtracting current cluster density $\rho_{i}^{c}$ to previous cluster density $\rho_{i-1}^{c}$

$$\rho_{i}^{diff}=\rho_{i}^{c}-\rho_{i-1}^{c}$$ (13)

From here we obtain the density to height ratio.


[ρh]i=ρidiffnch (14)

Next, the collective features related to density to volume ratio (denoted by *ε*) is defined as:

ε=[Vi−1c,Vic,Vidiff,[ρV]i] (15)

For *i* = 1,2,…*t*. The total number of parts ***t*** set to be 10 as default as shown in Eq (11)

$$i=\frac{z_{max}-z_{min}}{n}$$ (16)

where *i* represents the number of parts. The volume difference $V_{i}^{diff}$ is acquired by subtracting current cluster volume $V_{i}^{c}$ to previous cluster volume $V_{i-1}^{c}$

$$V_{i}^{diff}=V_{i}^{c}-V_{i-1}^{c}$$ (17)

The density difference is acquired as shown in (13). Therefore, the density to volume ratio is denoted by:

[ρV]i=VidiffVic (18)

**Fig 4:**
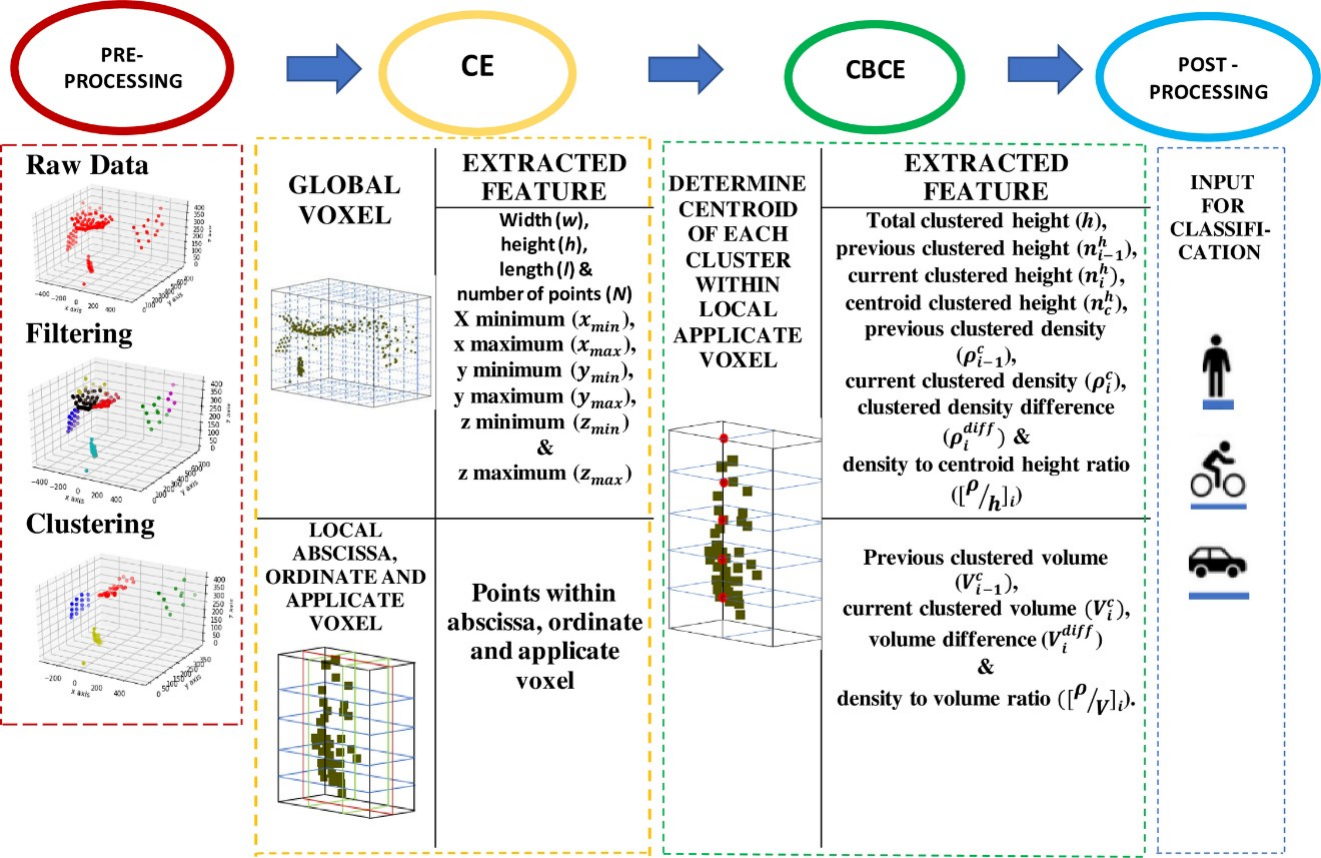
Flowchart of the proposed feature extraction method through CE-CBCE.

The flow chart of the proposed CE-CBCE feature extraction method can be seen in Fig 4.

Summary of features extracted are shown in Table 2 with its dimensional count and feature description.

**Table 2:** Summary of features extracted.

Feature name	Variable abbreviation	Dimensional Count	Feature Description
**Clustered Extraction (CE)**	*α*	4	**Width (*w*), height (*h*), length (*l*) & number of points (*N*)**
	*β*	120	**Points within abscissa, ordinate and applicate voxel**
	*γ*	6	**X minimum (*x***_***min***_ **), x maximum (*x***_***max***_ **), y minimum (*y***_***min***_ **), y maximum (*y***_***max***_ **), z minimum (*z***_***min***_ **) & z maximum (*z***_***max***_ **).**
**Centroid based Clustered Extraction Method (CBCE)**	*δ*	8	**Total clustered height (*h*), previous clustered height ($n_{i-1}^{h}$), current clustered height ($n_{i}^{h}$), centroid clustered height ($n_{c}^{h}$), previous clustered density ($\rho_{i-1}^{c}$), current clustered density ($\rho_{i}^{c}$), clustered density difference ($\rho_{i}^{diff}$) & density to centroid height ratio ([ρh]i)**
	*ε*	4	**Previous clustered volume** ($V_{i-1}^{c}$), current clustered volume $\left(V_{i}^{c}\right)$, volume difference ($V_{i}^{diff}$) & density to volume ratio ([ρV]i)

From the extracted features, classifications are done with 75%-25% split of training and testing data. Training aimed to decrease the model loss function value against training data as each step was processed. Model performance was indicated and measured through improvements in accuracy of the model against the test dataset [51].

The accuracy of the classifications is calculated as follows:

$$\frac{\sum_{i=1}^{k}\frac{{TP}_{i}+{TN}_{i}}{{TP}_{i}+{TN}_{i}+{FP}_{i}+{FN}_{i}}}{k}$$ (19)

With *k* representing the total number of class, *TP*_*i*_ as the true positive, *TN*_*i*_ as the true negative, *FP*_*i*_ as false positive and *FN*_*i*_ as false negative; for *i* = 1,2,3,…*k*.True positive is when the model correctly predicts the positive class, and a false negative is recorded when a class is incorrectly predicted to be negative. False positive occurs when a class is incorrectly predicted to be positive, and true negative is considered when the model correctly predicts the negative class. However for multiclass classification such as our case [48], true positive occurs only when the right class is correctly predicted, and similarly for false negative etc.: they all depend on the class.

## Results and discussions

Post CE and CBCE extraction, the collective features are optimized with kNN, DT and CNN. The method proposed is compared with three feature extraction methods which are the region of interest (ROI) [32, 52], feature vector (FV) [19] and multiple feature extraction (MFE) [30].

The chosen comparative method is considered as it similarly handles sparse point cloud, has low computing cost, employs geometrical features and runs on real-time execution. For ROI, the authors proposed taking width (*w*), length (*l*), height (*h*), width difference (Δ*w*) and length difference (Δ*l*) as the extracted features for classification. So, the complete geometric feature is Δ*G* = [*w*,*l*,*h*,Δ*w*,Δ*l*].

The second comparison method is the feature vector (FV) introduced by Wang et al. [19]. The feature extracted includes 2D covariance matrix in 3 zones, 2D histogram for x-y plane and 2D histogram for y-z plane. The total dimensional count amounts to 175 features.

The third comparison method is proposed by Tian et al. [30], with multiple feature extraction which includes point count (*N*), point density (*ρ*), voxel centroid (*μ*), point variance (*σ*^2^), point covariance (${\bar{\sigma }}^{2}$), point eigenvector (*ν*), point eigenvalue (*γ*), surface curvature (*k*) and divergence degree (*F*). The final feature extraction of MFE includes 27-dimensional count, with 9 counts from point eigenvector, 3 counts each from voxel centroid, point variance, point covariance, point eigenvalue and divergence degree, and finally a single dimension count from point count, point density and surface curvature.

The results obtained are then compared in terms of accuracy, precision, recall and F1-score. k-Fold Cross-validation is performed on the classifiers to select model parameters which best fit our data. Table 3 shows the complete hardware and software configurations for the experiments conducted. The simulation was done on an Intel (R) Core (TM) i7-5500U CPU @ 2.40GHz, 8Gb RAM and 64-bit operating system.

**Table 3:** Complete hardware and software configurations.

Configuration		Function
**Hardware Configuration**	Lidar Lite V3	Scanning LiDAR point cloud
	Arduino Nano/Uno	Microcontroller
	LiPo Battery 2200mah	Power supply
	FS5109 Servo Motor x 2	Moving actuating LiDAR
	DC motor x 2	Enable mobile robot movement
	L298N Motor Driver	Controlling DC motor
	Arduino XBee	Wireless data transmission
	Tyre x 2	Moving compartments
	Acrylic sheet frame	Frame body parts
	Servo Bracket	Servo placement
	Capacitor	Power supply smoothing
	Connecting wires	Electricity connections
**Software configuration**	Google Colaboratory	Processing and computing in central computer
	Jupyter notebook	Processing and computing in central computer
	Meshlab 2016.12	Point cloud visualisation
	Arduino 1.6.8	Processing and computing in the mobile robot

### Classification experiments

The experiment was done on an Intel(R) Core (TM) i7-5500U CPU @ 2.40GHz, 8Gb RAM and 64-bit operating system.

The results of our experiment are represented in Table 4. The "Parameter" column shows the varying parameters to be determined to achieve the best configuration for each optimization algorithm. The parameters refer to the number of nearest neighbors for k-NN, the maximum depth for DT and the number of layers for CNN. For DT and k-NN the parameters are set in the range of 1 until 30. For CNN, the number of hidden layers is ranged between 1 to 10, with the batch size of 10, 1000 the number of epochs, and an activation function of Rectified Linear Units (ReLUs) and Softmax function. For the proposed method, initially the CE and CBCE methods are implemented separately, before combining both collective features to show the improved performance.

**Table 4:** Accuracy results comparison of ROI, FV, MFE, CE, CBCE and CE-CBCE.

Method	Parameter	Value	Accuracy (%)					
			FV [19]	ROI [52]	MFE [30]	CE	CBCE	CE-CBCE
**k-NN**	**Nearest Neighbour**	5	70	85	87	90	89	90
		10	68	82	85	90	87	88
		15	67	80	82	88	85	86
		20	66	81	80	89	83	84
		25	66	81	79	89	83	84
		30	63	79	76	88	80	81
**DT**	**Max Depth**	5	74	77	82	86	80	87
		10	76	86	85	93	85	91
		15	77	84	86	92	87	89
		20	76	84	87	90	87	88
		25	78	84	87	90	86	89
		30	77	85	86	91	74	87
**CNN**	**No of Hidden Layers**	2	64	67	88	90	89	91
		4	62	80	91	93	89	95
		6	64	76	87	93	91	97
		8	67	69	90	91	93	95
		10	66	79	90	92	89	92

Table 4 shows the comparison of the results in terms of accuracy for ROI, FV, MFE, CE, CBCE and fusion of CE-CBCE combined. The selected results displayed are with the increment of 5 for k-NN and DT parameters and 2 for CNN parameter.

From Table 4, the general accuracy overview results in over 65% accuracy for all feature extraction methods. In terms of consistency, the CE method scores the highest mean accuracy at 88.3%, followed by CE-CBCE (85.4%), CBCE (84%), MFE (83.2%), ROI (79%) and finally FV with 69.3%.

**Fig 5:**
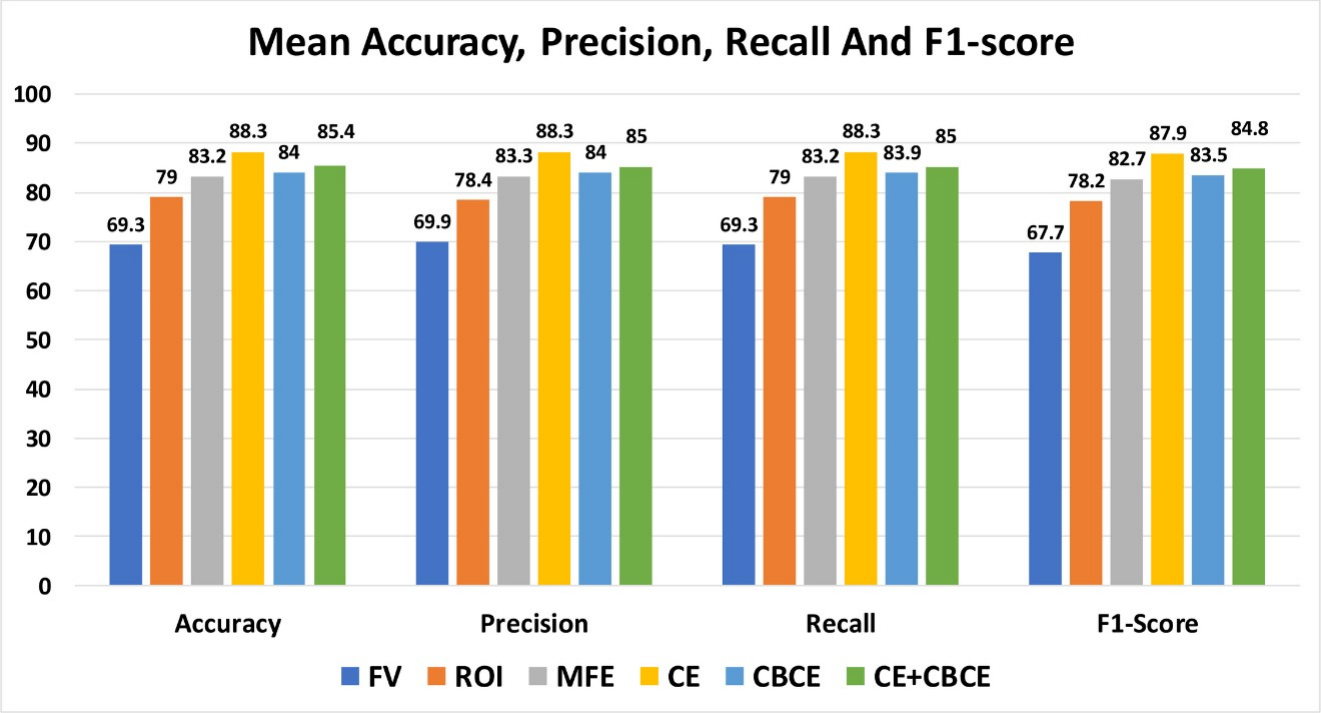
Accuracy, precision, recall and F-1 score of all feature extraction methods across each optimization algorithms.

A complete comparison in terms of mean accuracy, precision, recall, and F1-score can be seen in Fig 5. In general, the CE method prevails in terms of accuracy, precision, recall and F1-score across all optimization methods of k-NN, DT and CNN.

**Fig 6:**
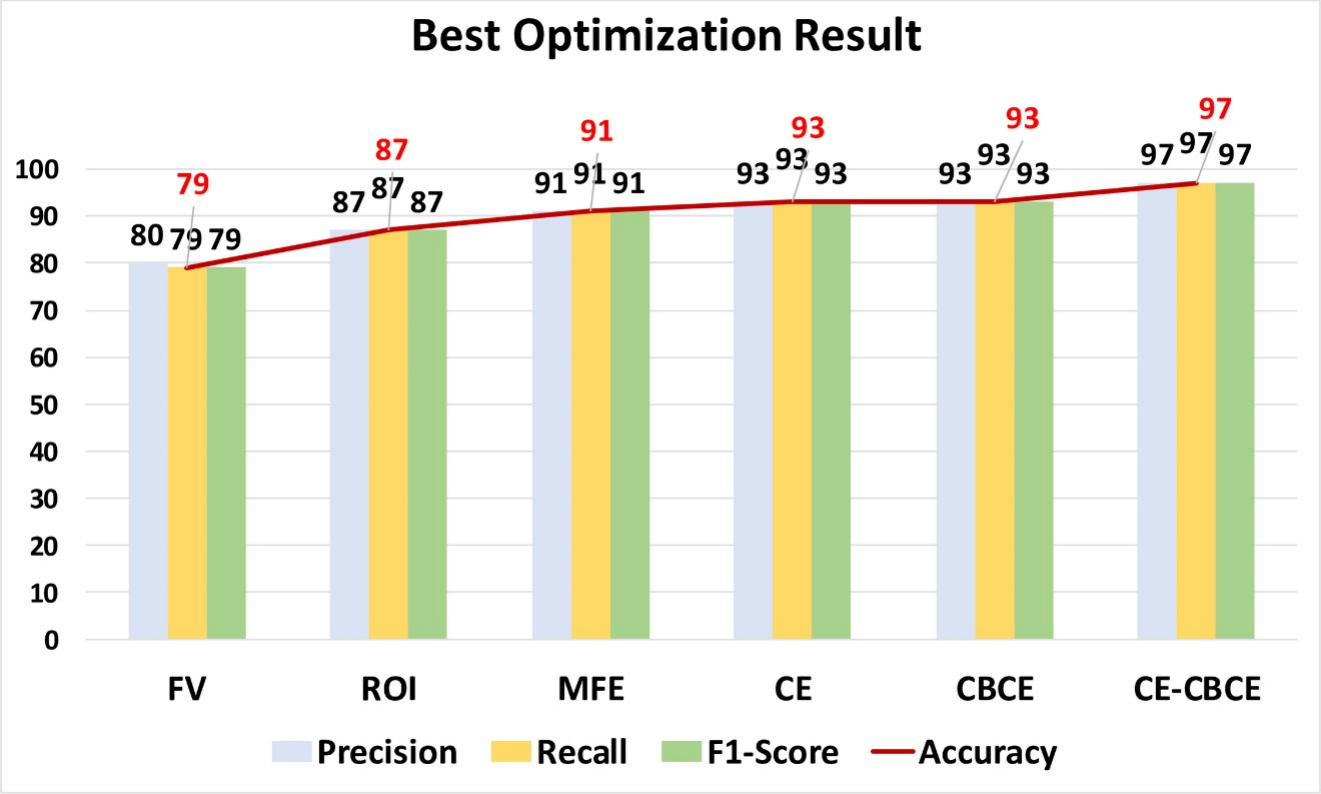
Best optimization result for each feature extraction method.

After analysis of the statistics across all optimization methods, a particular examination of each best performing optimization parameter is done. Specifically, the CE-CBCE method optimized by CNN with 6 hidden layers recorded best accuracy for object recognition and classification at 97% detection. This is followed by both the CE and CBCE method optimized with k-NN with the k-value of 1 and CNN with 8 hidden layers, respectively. Both methods recorded accuracy of 93% detection. The rest of the feature extraction methods achieved 91% for MFE, 87% for ROI and 79% for FV. Fig 6 shows the best optimization results in terms of precision, recall, F1-score and accuracy for each feature extraction method.

The best parameter choice for each method can be seen in Table 5. The precision, recall and F1-score for each individual class of human, motorcyclists and cars are presented with its final accuracy.

**Table 5:** Best result with its method of optimization and parameter for each class of objects.

Feature Extraction	Optimization		Human			Motorcyclist			Car			Acc
	Method	Parameter	Pre	Re	F1	Pre	Re	F1	Pre	Re	F1	
**ROI**	**kNN**	3	91	98	94	87	81	84	84	85	84	87
**FV**	**DT**	9	80	95	87	84	63	72	76	83	79	79
**MFE**	**CNN**	4	95	94	95	88	89	89	90	90	90	91
**CE**	**kNN**	1	100	96	98	87	91	89	92	91	92	93
**CBCE**	**CNN**	8	98	95	96	90	91	90	92	93	93	93
**CE-CBCE**	**CNN**	6	100	96	98	93	98	96	97	96	96	97

**Fig 7:**
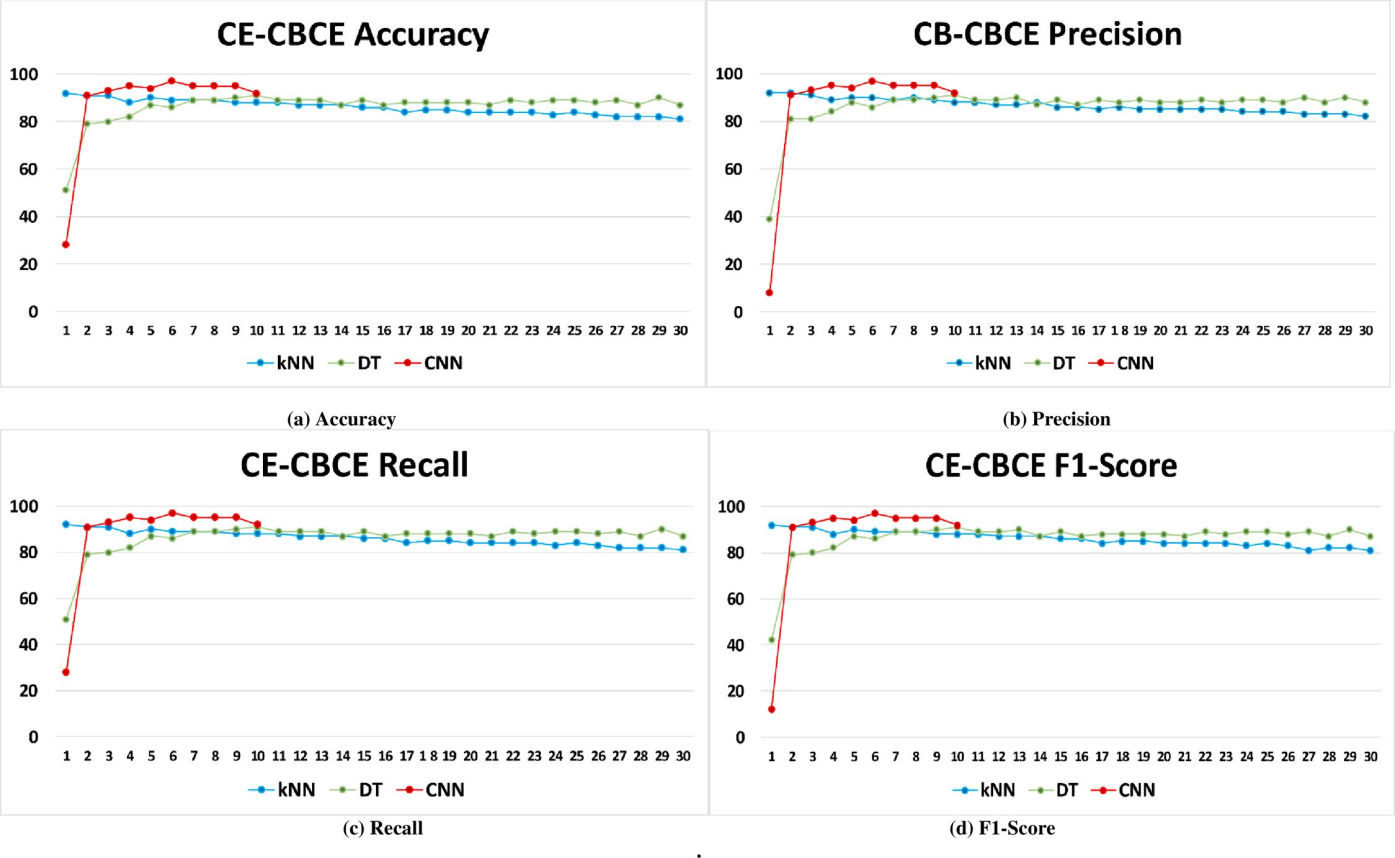
Full results of the proposed extraction CE-CBCE method across all optimization techniques.

Full results of CE-CBCE across all optimization techniques are shown in Fig 7 on the following page. Results achieved across all parameters are in the graphs within said figure.

As satisfying results are achieved for object recognition amongst 3 classes of objects, another experiment with added difficulty is conducted. The aim of classifying within 3 classes of objects remains the same, however this time around the output of the prediction includes the pose of subject. Therefore, for each detected object, the direction pose needs to be predicted whether it is facing front, right, back of left side towards the mobile robot. The same number of samples have been provided as the input, with 300 samples for each class and 100 samples for each orientation. Table 6 shows the best results obtained from each feature extraction methods.

**Table 6:** Object prediction with pose detection.

Feature Extraction	Optimization		Human (%)				Motorcyclist (%)				Car (%)				Acc (%)
	Method	Parameter	Front	Right	Left	Back	Front	Right	Left	Back	Front	Right	Left	Back	
**ROI**	**kNN**	**1**	**89**	**95**	**90**	**100**	**57**	**65**	**6 8**	**71**	**47**	**43**	**54**	**69**	**70**
**FV**	**DT**	**14**	**82**	**68**	**80**	**92**	**43**	**62**	**36**	**50**	**26**	**47**	**62**	**76**	**60**
**MFE**	**CNN**	**10**	**88**	**93**	**86**	**96**	**66**	**55**	**61**	**68**	**58**	**81**	**83**	**97**	**77**
**CE**	**CNN**	**5**	**92**	**100**	**100**	**100**	**81**	**83**	**81**	**76**	**68**	**57**	**67**	**93**	**81**
**CBCE**	**CNN**	**4**	**81**	**89**	**85**	**88**	**81**	**86**	**65**	**76**	**63**	**57**	**78**	**97**	**78**
**CE-CBCE**	**kNN**	**1**	**96**	**100**	**100**	**96**	**75**	**71**	**89**	**70**	**67**	**79**	**74**	**85**	**82**

**Fig 8:**
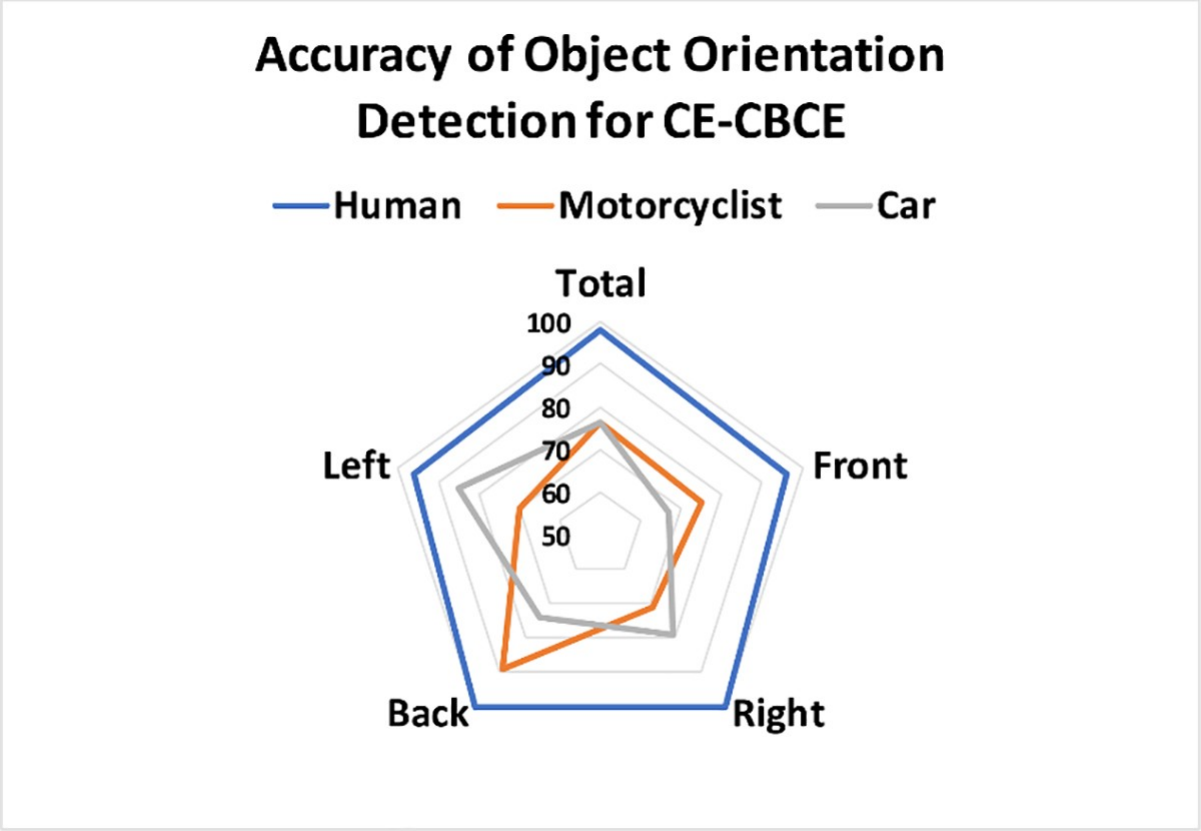
Accuracy results of CE-CBCE for each orientation.

In the Table 6, excellent results for the object’s orientation are recorded. The combination of CE-CBCE method achieved accuracy of 82%, followed by CE, CBCE, MFE, ROI and finally FV methods. Especially for easily perceived poses for human subjects facing right or left, CE-CBCE and CE methods both recorded 100% detection rate. The radar chart in Fig 8 shows the accuracy of CE + CBCE method for each object with varying pose orientation.

## Conclusions

**Fig 9:**
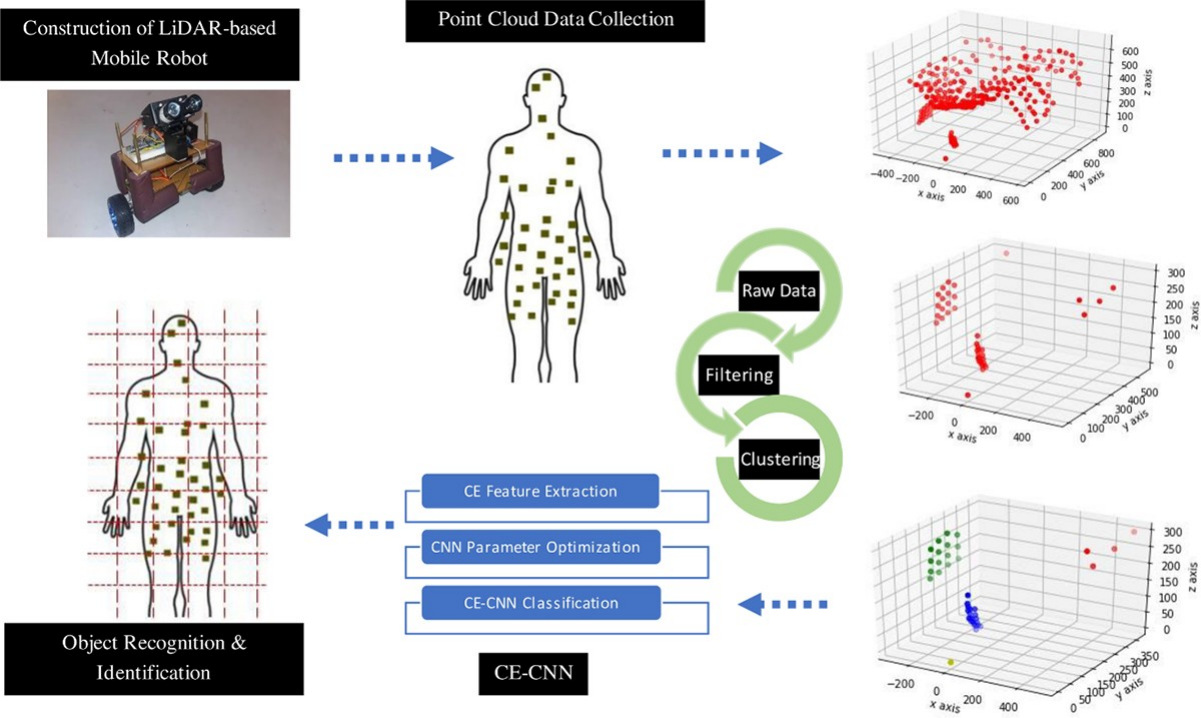
Flow of the proposed feature extraction technique for object detection.

In this research, we proposed a novel feature extraction for sparse LiDAR point cloud object recognition. Indoor and outdoor data were collected, with different backgrounds for better simulation of varying surroundings. We also analysed the performance of our feature extraction method with different classes of objects in varying pose orientations. The result shows a promising achievement with a sparse LiDAR point cloud. The flow of the research proposed can be seen in Fig 9. The process started form hardware development, before moving into genuine data collection, preprocessing, proposed feature extraction method, classification algorithms and finally object classification.

As the proposed method targeted sparse LiDAR point cloud input, its performance on high density data remains to be explored. High density, compact point clouds such as autonomous vehicle and airborne LiDAR are often associated with large scale mapping and varying elevation scanning. A larger number of classes for object detection could also pose a challenge as they tend to be less distinctive in terms of density and point cloud distribution.

For future research, position tracking can be implemented on top of the object recognition and classification. Especially for a safety-critical system where accuracy is of utmost importance, safety features should be a main concerning issue. A fail-safe mechanism in place to override the controls in case of a malfunction could be implemented, with a warning system which alerts the user during detection of a faulty device to allow human intervention. Additional elements or subjects of detection can also be added to further test and improve the reliability of the system.
